# Genetic mapping of loci affecting embryogenic callus formation and in vitro regeneration in cereals and leguminous crops

**DOI:** 10.18699/vjgb-25-54

**Published:** 2025-07

**Authors:** Е.К. Potokina, A.S. Sushchenko

**Affiliations:** Skolkovo Institute of Science and Technology (Skoltech), Moscow, Russia; Skolkovo Institute of Science and Technology (Skoltech), Moscow, Russia

**Keywords:** plants, in vitro, genotype-dependent regeneration, recalcitrance, genetic control, QTLs of morphogenetic traits, растения, in vitro, генотип-зависимая регенерация, рекальцитрантность, генетический контроль, QTL морфогенетических признаков

## Abstract

Recalcitrance is defined as the inability of plant species or individual genotypes to effectively regenerate and/or to be transformed in in vitro culture, and is the most significant limitation for genome editing of agricultural crops. To develop protocols for genotype-independent transformation and regeneration of cultivated plants, knowledge of the genetic factors that determine recalcitrance in various plant species under in vitro conditions is required. Their search by classical QTL mapping in populations segregating for callus formation efficiency, regeneration, and transformation is considered a complex and labor-intensive process due to a specific nature of the analyzed phenotypes and a strong genotype-environment relationship. The article provides an overview of the methodology, prospects, and most outstanding achievements of “forward” genetics in identifying genetic determinants of recalcitrance in the most popular and at the same time most difficult to work with in vitro cereal and legume crops. Examples of genetic mapping and successful cloning of genes responsible for various aspects of recalcitrance in cereals are discussed. Thus, it was found that the formation of rapidly proliferating type II embryogenic callus in maize is determined by increased expression of the Wox2a gene. The Koshihikari rice variety, popular in Japan, poorly regenerates in vitro due to impaired nitrate metabolism, since it has a low expression level of nitrite reductase (NiR), which converts nitrite into ammonia. Callus browning, which occurs among many plant species and leads to a decrease in regenerative capacity and even to plant death, in rice varieties (Oryza sativa ssp. indica) depends on the expression level of the Browning of Callus1 (BOC1) gene, which encodes the SRO protein (Similar to RCD One), regulating the plant response to oxidative stress. Similar studies on mapping loci for somatic embryogenesis traits in soybean have revealed major QTLs explaining 45 and 26 % of phenotypic variation. Studies on genetic mapping of loci affecting the efficiency of regeneration and embryogenesis in recalcitrant plant species have obvious prospects due to the emergence of annotated reference genomes, high-throughput genotyping and high-resolution genetic maps

## Abstract (ru)

Рекальцитрантность определяется как неспособность видов или отдельных генотипов растений к эффективной регенерации и/или трансформации в культуре in vitro и представляет собой самое существенное ограничение для геномного редактирования сельскохозяйственных культур. Для разработки протоколов генотип-независимой трансформации и регенерации культурных растений необходимы знания о генетических факторах, детерминирующих рекальцитрантность у различных видов растений в условиях in vitro. Поиск их путем классического картирования QTL для признаков эффективности каллусообразования, регенерации, трансформации в расщепляющихся популяциях считается сложным и трудоемким процессом из-за специфичной природы анализируемых фенотипов и сильной взаимосвязи «генотип – среда». В статье приводится обзор методологии, перспектив и наиболее ярких достижений «прямой» генетики в идентификации генетических детерминант рекальцитрантности у самых востребованных и одновременно наиболее трудных для работы in vitro зерновых и бобовых культур. Приведены примеры генетического картирования и успешного клонирования генов, отвечающих за разные аспекты рекальцитрантности у злаков. Так, установлено, что формирование быстро пролиферирующего эмбриогенного каллуса II типа у кукурузы определяется повышенной экспрессией гена Wox2a. Популярный в Японии сорт риса Koshihikari плохо регенерирует в культуре in vitro из-за нарушенного метаболизма нитратов, так как отличается низким уровнем экспрессии нитритредуктазы (NiR), преобразующей нитрит в аммиак. Побурение каллуса, встречающееся среди многих видов растений и приводящее к снижению регенерационной способности, у сортов риса (Oryza sativa ssp. indica) зависит от уровня экспрессии гена Browning of Callus1 (BOC1), который кодирует белок SRO (Similar to RCD One), регулирующий реакцию растения на окислительный стресс. Аналогичные работы по картированию локусов для признаков соматического эмбриогенеза у сои позволили обнаружить мажорные (major) QTL, объясняющие 45 и 26 % изменчивости признака. Исследования по генетическому картированию локусов, влияющих на эффективность регенерации и эмбриогенеза у рекальцитрантных видов растений, имеют очевидные перспективы в связи с появлением аннотированных референсных геномов, высокопроизводительного генотипирования и генетических карт с высоким разрешением.

## Introduction

In recent years, significant achievements in the field of plant genome editing have contributed to the rising number of new varieties and clones with introduced mutations that are of interest for agricultural practice. In 2017, 20 crops were reported to be improved using CRISPR/Cas9 technology (Ricroch et al., 2017); by 2020, genome editing had been applied to 40 crops in 25 countries to improve their yield and resistance to biotic and abiotic stresses (Menz et al., 2020). However, if we classify the current status of crop genome editing projects into five sequential stages of development and implementation: (1) discovery; (2) proof of concept; (3) early development; (4) advanced development; (5) commercialization, then by 2022, most such developments were in the “early development” stage, and only rice genome editing was categorized as “advanced development” (Pixley et al., 2022). The lack of new commercial varieties improved using CRISPR/Cas9 is explained not only by legal restrictions, but also by the fact that in most cultivated plant species, only a small number of tested genotypes are capable of regular and efficient development of embryogenic and regenerative tissues under standard in vitro conditions (Nam et al., 1997; Salvo et al., 2018; Nivya, Shah, 2023; Nagle et al., 2024).

Recalcitrance in vitro is defined as the inability of plant cells, tissues and organs to respond to manipulations in tissue culture (Benson, 2000). Recalcitrance concerns not only regeneration but also transformation efficiency: sometimes successfully regenerating cells fail to be transformed using Agrobacterium, and vice versa, successfully transformed cells fail to regenerate. The failure of plants to effectively regenerate and/or transform represents the most significant limitation for transgenesis and genome editing in crops (Altpeter et al., 2016).

The traditional approach to overcoming plant recalcitrance in vitro is to work on optimizing external factors, including the composition of the basal medium, pH, lighting conditions, types of explants, etc. In most cases, the plant cell development program is changed by adding growth regulators (auxins and cytokinins) to the medium. In this case, the choice of growth regulators, their arrangement and time of exposure are usually determined empirically for each species and are often adjusted for each genotype (Altpeter et al., 2016). At the same time, research aimed at identifying the genetic and epigenetic mechanisms controlling somatic embryogenesis and callus formation has made it possible to manipulate these processes more finely using hormonal signals (Maren et al., 2022).

Significant progress in the technology of transformation of monocots and recalcitrant dicot species has been achieved by manipulating so-called “morphogenic genes” to reprogram somatic cells to initiate embryogenesis. Such morphogenic genes include, in particular, key regulators of the development and determination of meristematic cells, such as Baby Boom (BBM), Wuschel (WUS) and Wuschel- Related Homeobox (WOX ) (Chen Z. et al., 2022).

The development of “reverse” genetics methods has led to the fact that today several dozen such morphogenic genes that regulate the growth and development of plants in vitro are known. The term “fine-tuning” has appeared in the scientific literature, meaning precise adjustment of the expression level of key morphogenic genes, ensuring successful transformation and regeneration of plants (Maren et al., 2022). For example, applying such adjustments to the expression of the BBM and WUS2 morphogenic genes, it was possible to induce somatic embryogenesis and regenerate fertile transgenic plants of corn, sorghum and sugarcane from calli of an immature embryo (Lowe K. et al., 2016). In this particular study, low expression of the WUS2 gene under the low-efficiency monocot nopaline synthase promoter (Nos:ZmWUS2) was combined with increased expression of the BBM gene under the “strong” maize ubiquitin promoter (ZmUbi:ZmBBM). As a result of such tuning of the expression of two morphogenic genes in maize, it was possible to obtain transformed fertile plants from 40 % of the calli of the inbred line Pioneer PHH5G, which had previously not been transformed using bioballistics or agrobacterium. For maize, 53 potential morphogens have been described to date that affect the efficiency of regeneration and transformation, and manipulation of the most effective of them – transcription factors ZmWIND1 and ERF/AP2 – allows increasing the frequency of callus formation by 60.22–47.85 % and transformation by 16.56– 37.2 %, depending on the genotype (Jiang et al., 2024).

Dozens of similar examples of successful manipulation of morphogenic genes expression for efficient transformation of agricultural crops (corn, rice, wheat, triticale, barley, sorghum, soybean, beet, rapeseed, tomato, pepper, potato, turnip, grapes) (Chen Z. et al., 2022) indicate that the development of protocols for genotype-independent transformation and regeneration of crop plants may eventually become not so much an art as a technology. However, this requires knowledge of the genetic factors influencing somatic embryogenesis, the formation of embryogenic callus and the regeneration of various types of cultivated plants in vitro. The search for them using the classical QTL mapping for traits of callus formation efficiency, regeneration, transformation in segregating populations is considered a complex and labor-intensive process due to the specific nature of the analyzed phenotypes and the strong genotype-environment relationship affecting the plant’s responsiveness to manipulations in vitro (McFarland et al., 2023).

The purpose of this article is to review the methodology, prospects and most impressive achievements of forward genetics in identifying genetic determinants of recalcitrance in the most popular and at the same time most difficult to work with in vitro cereal and legume crops.

## Mapping of loci that negatively affect the regeneration of cereal crops

The issues of low regenerative capacity of explants in vitro and genotype-dependent transformation of cereals have received the most attention in the literature because these crops provide the majority of calories consumed by humanity (Chen Z. et al., 2022). In many important cereals such as rice, wheat, barley and maize, embryogenic regenerating callus cultures have been limited to a few genotypes for several decades, restricting the possibilities of breeding these crops using biotechnology (Kausch et al., 2021).

A good example is the search for loci that determine the genotype-specific regenerative capacity of inbred maize lines (McFarland et al., 2023). Several maize genotypes, namely H99 (Duncan et al., 1985), B104 (Frame et al., 2011) and LH244 (Altschul et al., 1990), are capable of forming slow-growing, compact, highly heterogeneous embryogenic type I callus in vitro. From a biotechnological point of view, it is much preferable to work with embryogenic callus type II, which is looser, proliferates rapidly, has high embryogenic and regenerative capacity. This type II callus was identified in a single inbred line A188 more than 40 years ago (Green, Phillips, 1975). Since then, despite an active search for new maize lines suitable for manipulation in vitro, highly embryogenic type II callus has remained a specific attribute of a single genotype A188 and its derivatives (McFarland et al., 2023). Numerous attempts to optimize the composition of the culture medium have allowed some increase in the efficiency of callus formation and regeneration of fertile transgenic plants (Gordon-Kamm et al., 1990). However, effective regeneration of transgenic maize plants was achieved only for a few genotypes that were of little interest from an agronomic point of view (McFarland et al., 2023).

In response to the challenge from practical selection, a breeding program was initiated in the 1970s to obtain “culturable” lines of maize by crossing the unique line A188 with the inbred line B73, which was valuable from a selection point of view, but recalcitrant in in vitro culture (Russell, 1972). As a result of a series of recurrent backcrosses, lines were obtained with an introgressive fragment A188 on chromosome 3, which determines regeneration ability (Armstrong et al., 1992). Another decade later, through additional crosses, it was possible to obtain “culturable” lines that inherited only 15 % of their genome from A188 (Lowe B.A. et al., 2006). At this stage, it was still not possible to identify the causative gene, but molecular markers linked to it were identified. With the publication in 2009 of the reference genome of the B73 maize line, as well as the advent of high-throughput genotyping tools (Illumina 55k Maize SNP Chip), it became possible to conduct more accurate QTL mapping for the trait “ability to form embryogenic callus in in vitro culture”, using the same material from crossing contrasting parents A188 and B73, converted into almost isogenic and double haploid lines. As a result, the desired interval on chromosome 3 was narrowed to 3,035 Gb (Salvo et al., 2018).

In 2023, following a series of additional backcrosses and with the help of the annotated reference genome of the parental line B73, 93 potential candidate genes were identified. Based on the results of their transcription analysis using the RNAseq method, three most likely candidates were identified, the increased expression of which in explants was achieved using vectors with a “strong” maize ubiquitin promoter (ZmUbi1), and this made it possible to assess the effect of the expression level of potential candidate genes on the development of embryogenic callus. As a result, the Wox2a gene was identified for the first time, underlying the QTL for the ability to form embryogenic callus, which was mapped in the population of offspring from crossing the inbred maize lines A188 and B73. The differences in the structural part of the Wox2a gene in the contrasting parental genotypes were minimal, but the promoter region coincided only by 69 %. It was concluded that the increased expression of the Wox2a gene could be the cause of the formation of type II embryogenic callus in the A188 line (McFarland et al., 2023).

Another example of successful cloning of a gene affecting regeneration and embryogenesis has been described in rice, for which an efficient in vitro system had previously been developed in model varieties such as Nipponbare (Oryza sativa ssp. japonica) and Kasalath (O. sativa ssp. indica). However, many leading rice varieties used for food production in Japan, such as the Koshihikari variety, had low regeneration capacity of mature embryo in vitro, which was a serious obstacle to the efficient production of transgenic plants (Nishimura et al., 2005). The recalcitrant genotype Koshihikari formed calli that invariably turned brown in tissue culture and never gave rise to green shoots. The contrasting genotype Kasalath, used for crossing with Koshihikari, on the contrary, was distinguished by the ability to form viable calli from which new shoots were successfully regenerated.

The trait “regeneration ability” (the number of shoots regenerated from the callus) was mapped using a population of 99 progeny of the BClFl generation using 262 PCR markers regularly distributed over 12 rice chromosomes. Four significant QTLs were mapped on chromosomes 1, 2, 3 and 6, and at all these loci, Kasalath alleles had a positive effect on the regeneration ability of plants. The QTL on chromosome 1 showed the greatest effect, was designated as PSR1 (Promoter of Shoot Regeneration 1) and was subjected to fine map-based cloning using 3,800 recombinants of the BC3F2 generation. The desired chromosomal interval was narrowed to 50.8 kb, but to identify the PSR1 gene, it was necessary to construct a BAC (Bacterial Artificial Chromosome) library from the genomic DNA of the Kasalath variety, in which the BHAL15 clone covering the desired region of the genome was identified. Next, several sequences covering possible candidate genes were subcloned from BHAL15 and used to transform calli of the recalcitrant Koshihikari genotype. One of these subclones, 12.2 kb in size, overlapping the sequence of the candidate gene NiR encoding ferredoxin-nitrite reductase, restored the regenerative capacity of Koshihikari calli and, on this basis, was identified as the causative gene for the trait in question.

Comparison of the NiR gene sequences in the Koshihikari and Kasalath varieties revealed several SNPs and InDels, especially in the promoter region of the gene. The mutations found in the structural part of the gene led to only two conservative amino acid substitutions in the encoded protein; on the other hand, the expression level of this gene in the recalcitrant Koshihikari variety was 2.5 times lower than in the Kasalath variety. It is also interesting that in the Koshihikari variety, in addition to the full-length NiR transcript, a transcript with a retained (third) intron was also detected. Reduced NiR expression in Koshihikari apparently led to a disruption of nitrate metabolism in this rice variety, since in this metabolic pathway nitrate reductase catalyzes the reduction of nitrates to nitrites, and nitrite reductase (NirBD) converts nitrite to ammonia. Disrupted nitrate metabolism probably caused the low embryogenic capacity of Koshihikari rice calli.

Another example of positional mapping of recalcitrance loci in rice concerns the callus browning effect in vitro, which is typical to the widespread varieties of O. sativa ssp. indica (Zhang K. et al., 2020). Callus browning occurs in many plant species and results in reduced regenerative capacity, poor in vitro growth, and plant death (He et al., 2009). The use of antioxidants, adsorbents, low salt concentrations and growth regulators can reduce the effects of callus browning to some extent, but there is no universal solution to this problem (Zhang K. et al., 2020).

To search for loci responsible for callus browning in rice, a population of offspring was created by crossing the YJCWR genotype of the wild species O. rufipogon Griff., which is relatively resistant to callus browning (donor), and the elite variety Teqing (O. sativa ssp. indica) (recipient). In the population of hybrids, the YIL25 progeny line was isolated, in which the callus browning frequency were significantly lower than in Teqing, while in the YIL25 line, introgressions from the donor parent YJCWR were found on chromosomes 2, 3, and 5.

Backcrossing of the YIL25 line with the recipient parent Teqing yielded a population of 198 BClF2 lines, which were genotyped using microsatellite markers and used to map the QTL for the callus browning trait. The QTL mapped on chromosome 3 explained 14 % of the observed variability. To map this QTL at a higher resolution, a fraction of BClF2 lines heterozygous at the QTL interval were selfed, resulting in 6,377 recombinants. Their genotyping using SNP markers allowed to narrow the QTL interval to 18.6 kb, in which only one coding sequence, LOC_Os03g12820, annotated with the reference genome (The Rice Genome Annotation Project Database) was identified. Comparison of the sequence of this gene in the parental lines Teqing and YIL25, which were contrast in the analyzed trait, did not reveal polymorphism in the structural part of the gene, but a deletion of 337 bp, 1 bp and three SNPs were found in the promoter region of the Teqing variety. The sequence LOC_Os03g12820 was thus identified as a candidate gene Browning of Callus1 (BOC1), which affects callus browning.

A comparative analysis of the BOC1 expression level in the parental genotypes YIL25 and Teqing showed an almost twofold difference that reached its maximum values starting from the 21st day of callus cultivation and did not appear at earlier stages. Additional experiments on protoplasts with constructs representing various variants of mutations in the BOC1 promoter integrated into the pGreenII 0800-LUC vector made it possible to evaluate the effect of these mutations on the expression level of the luciferase reporter gene (LUC), and to establish that it is the insertion of 337 bp in the BOC1 promoter in the YIL25 genotype, resistant to callus browning, that significantly increases the expression level of this gene in callus culture. It was also found that the 337 bp insertion in the BOC1 gene promoter is a transposon (Tourist MITE), and the presence of this insertion not only reduces callus browning in rice varieties, but also increases the transformation efficiency by 2.5 times.

At least 16 scientific publications devoted to genetic mapping of QTL for recalcitrance traits in cereals were published between 2000 and 2020, but only 6 of them reported identified candidate genes (QTGs) as final results (see review by Lardon, Geelen, 2020). The identification of QTGs has not always involved the long and thorough process of positional mapping of genetic loci with multiple crosses and obtaining thousands of recombinants. For example, in barley, the only variety that can be transformed by agrobacterium is Golden Promise (Hisano, Sato, 2016).

To identify the loci that ensure the “cultivability” of this variety, Golden Promise was crossed with the recalcitrant genotype Haruna Nijo. A total of 3,013 immature embryos were isolated from F2 caryopses that were inoculated with Agrobacterium tumefaciens carrying a plasmid with a reporter gene for resistance to hygromycin HPT (Hygromycin PhosphoTransferase). Of the 3,013 inoculated explants, 293 formed calli on a selective medium, and 60 of these calli regenerated into full-sized plants (Hisano, Sato, 2016). DNA analysis of these 60 plants showed the presence of the HPT transgene, and the fact that these plants regenerated from callus indicated that they inherited alleles from Golden Promise at loci critical for transformation and regeneration processes that the parent Haruna Nijo did not possess. Such TFA (transformation amenability) loci were identified on chromosome 2 (TFA2, TFA3) and chromosome 3 (TFA1), and the confidence interval of each of these QTLs was, on average, 40 centiMorgan (cM).

The next step was to answer the question of whether the genes of the transcription factors BBM and WUS2, already known for cereals, were localized in these large chromosomal intervals. For this purpose, the sequences of these genes in maize were used to search for homologous sequences in the barley genome, and it turned out that the barley BBM homolog falls into the TFA2 interval, and the WUS2 homolog falls into the TFA1 interval (Hisano et al., 2017). Although this study did not provide direct evidence on the effect of the BBM and WUS2 morphogenic genes on barley transformation efficiency, it was shown that introgression of chromosome regions 2 and 3, where these genes are localized, from the Golden Promise variety into the desired barley genotype helps to achieve a transformation level of 15.5–23.7 %, which can be considered a high result, since for the Golden Promise variety itself, the transformation efficiency is approximately 30 % (Hisano et al., 2017).

By using segregating populations from biparental crosses, it is possible to map loci that have different alleles only in a particular parent pair. With the advent of highthroughput genotyping, it has become possible to analyze the variability of traits associated with callus formation, regeneration, and somatic embryogenesis in large samples of unrelated genotypes using association analysis (GWAS, Genome Wide Association Mapping). So, 510 rice varieties were genotyped using several thousand SNPs identified by sequencing of this global sample with Illumina HiSeq 2000 (Zhang Z. et al., 2019).

An association study between SNPs and variability of three callus formation traits was performed: callus induction rate (CIR), callus induction speed (CIS), and time of the first callus appearance (T0). The first two traits (CIR and CIS) were correlated with each other (r2 = 0.881), while the correlation between T0 and the other two traits was low (–0.337 and 0.286). As a result, 88 significantly linked loci were identified: 33 loci for CIR, 31 for CIS, and 24 for T0, with the identified loci for the three traits not overlapping. Of the total 88 loci identified, 21 were detected within QTL intervals previously mapped for rice in other studies. Among others, candidate genes CRL1, OsBMM1, and OsSET1, which are orthologs of the LBD17/LBD29, BBM, and SWN genes in Arabidopsis, were proposed for callus induction frequency, where the role of these genes in callus formation has been previously demonstrated (Boutilier et al., 2002; Chanvivattana et al., 2004; Fan et al., 2012).

A similar genome-wide association study was conducted for the callus induction frequency trait on 110 rice (ssp. indica) accessions genotyped with 2,385,475 SNP markers (Kamolsukyeunyong et al., 2024). A unique feature of this study was that the trait was tested on three culture media: B5 (Gamborg), MS (Murashige–Skoog), and N6 (CHU). Notably, callus induction was affected by different loci on different media: for B5, such a QTL was mapped to chromosome 6, for MS, to chromosomes 2 and 6, on N6, callus induction was affected by four QTLs, two on chromosome 6, and two more QTLs on chromosomes 7 and 11. As in the previous study, the intervals of mapped QTLs did not overlap. This suggested that different genes may influence successful callus induction on different culture media. This noticeable example partly explains why QTL mapping for traits associated with callus induction and subsequent production of fertile transgenic plants is not a popular area of research today – there are too many factors that can affect the reproducibility of the results.

Another difficulty with association analysis is that GWAS allows one to identify interesting patterns related to the physiological mechanisms of the studied traits, but rarely results in the identification of causative genes. More often, genes in close proximity to a reliably associated SNP, or haplotypes in an identified region of a chromosome that differ in the manifestation of a trait, are proposed for subsequent detailed study.

## Mapping of QTLs for transformation efficiency and callus formation ability in legume crops

Widespread legumes of the tribe Phaseoleae (soybean, beans, cowpea), as well as pea and guar, are recalcitrant plants for in vitro culture, in contrast to some other legumes like Medicago and Lotus (Nivya, Shah, 2023). In the most popular crop, soybean, the efficiency of regeneration and transformation depends on a specific genotype and is acceptable for a few varieties, such as, for example, cv. Jack (Yang et al., 2009) or Williams, Williams79 and Williams82 varieties (Xu et al., 2022).

Two features of the behavior of legumes in vitro have been reported (Nivya, Shah, 2023). First, the regeneration efficiency can be quite high, but only in the absence of any transformation attempts that imply selective pressure. The reasons for this phenomenon are unknown, although optimization of transformation protocols may improve the situation (Bekalu et al., 2023). Second, most published experiments on legumes failed to demonstrate the inheritance of transgenes or edited genes in the T1 generation (Nivya, Shah, 2023). The reason for this low heritability of transgenes is most likely the chimerism of regenerants, in which the floral meristem cells giving rise to gametes remain untransformed, which ultimately also explains the low transformation efficiency.

Despite the obvious difficulties in overcoming the recalcitrance of legumes in vitro, studies on mapping morphogenic genes for this group of crop plants are very rare and not comparable in scale with similar studies in cereals.

For example, soybean is a popular object of reverse genetics of morphogenic genes (e. g., Chen F. et al., 2019; Hao et al., 2019), but only two studies on QTL mapping in biparental populations are known: for traits of somatic embryogenesis efficiency (Song et al., 2010) and callus induction (Yang et al., 2011). In the first study, a population of 126 recombinant inbred lines (RILs) from a cross between Peking (higher somatic embryogenesis capacity) and Keburi (low capacity) was generated. The population was genotyped with microsatellite markers and highly significant QTLs were mapped to chromosome C2(6) for the somatic embryogenesis frequency trait, explaining a very high percentage of the observed variability – 45.2 % (Satt307) and 25.97 % (Satt286). Such a significant effect may indicate the presence of so-called major QTLs in these intervals of chromosome 6 in soybean. Additional QTLs with less pronounced effects (6–7 %) were identified on chromosomes “H” and “G”, which correspond to chromosomes 12 and 18 according to the current nomenclature (https://www.soybase.org/about/lgs_and_chromosomes/). The second QTL mapping study for the callus induction frequency (CIF) trait was performed on a population of RILs from a cross between Kefeng (CIF = 0.69) and Nannong (CIF = 0.86). The most significant QTL for this trait was mapped to chromosome 14 (B2) and explained 16.6 % of the observed variability (Song et al., 2010).

An example of a genome-wide association study (GWAS) for in vitro culture-related traits in legumes has been published for peanut (Luo et al., 2024). To identify accessions with potential for regeneration, the authors compared the genotyping results of 353 peanut accessions from 26 countries with their ability to form embryogenic callus in vitro. Embryos isolated from sterilized seeds were placed on MS medium with vitamins, and explants were subcultured onto fresh medium every 4 weeks. It is reported that after the sixth passage, the physiological state of the callus began to stabilize, and the number of calli was recorded in the seventh, eighth, and ninth subculture each (T7, T8, and T9, respectively).

The analyzed trait, callus formation frequency, was designed as the ratio of the number of formed calli to the initial number of explants for each passage separately. 864,179 SNPs and 71,052 InDels were used for population genotyping. The correlation coefficient between the callus formation frequency in the T7, T8 and T9 subcultures varied from 0.56 to 0.61. As a result of the GWAS, 23 significantly associated SNPs were identified for the T7 subculture, 30 SNPs for T8, and 8 SNPs for T9. An important fact is that in this study, the same interval on chromosome 13 containing several SNPs associated with the trait was identified for all three passages. This fact may indicate the presence of a major QTL on chromosome 13 in peanut. The most reliable SNP in this region of the chromosome was identified in the gene encoding peroxisomal ABC transporter 1, which affects plant growth and development processes (Baker et al., 2015).

Another SNP from the same interval introduced an amino acid substitution in the Arahy.MIX90M gene encoding auxin response factor 19. The confidence interval on chromosome 13 also included SNPs in close proximity to the gene encoding the MYB transcription factor. In maize, genes of this family are involved in the formation of embryogenic callus via gibberellin signaling (Ge et al., 2016).

## Problems and prospects in searching for genetic determinants of recalcitrance in plants using QTL mapping

Mapping of QTLs controlling regeneration and transformation capacity is currently not a widely used research approach for overcoming in vitro recalcitrance in plants. The main reason is that mapped QTLs are often specific to particular experimental conditions, thus the results depend on the specific culture medium in which the explants are grown or on the specific stage of explant development at which the trait variability begins to manifest itself. Often, the identified QTLs reflect polymorphisms inherent only to a particular parental pair, and QTL mapping does not always result in the identification of a candidate gene.

Nevertheless, it is clear that the low regeneration and transformation efficiency of many crop species severely limits the potential of CRISPR-Cas technology to improve the agronomic performance of agricultural crops. Experience shows that knowledge of key genes encoding “global” transcription factors, the expression of which is capable of stimulating cell proliferation, makes it possible to solve this problem using biotechnological methods. An example of such an approach is the work of J.M. Debernardi et al. (2020), who created a construct expressing a chimeric protein combining the transcription factor Growth-Regulating Factor 4 (GRF4) of wheat and its cofactor GRF-Interacting Factor 1 (GIF1). GRF factors mediate interactions between proteins and between proteins and DNA, and GRF genes are highly conserved in angiosperms, gymnosperms, and mosses, indicating their fundamental importance for growth and development processes (Omidbakhshfard et al., 2015).

Expression of the chimeric GRF4–GIF1 protein in tetraploid wheat calli increased regeneration by 7.8 times and significantly reduced the time required to obtain regenerants. The same effect was observed when transforming triticale and rice calli with the same GRF4–GIF1 construct (Debernardi et al., 2020), as well as in experiments with barley (Timonova et al., 2023). J.M. Debernardi et al. (2020) also showed that homologs of the wheat GRF4– GIF1 genes expressed in the epicotyl of Carrizo citrus (a hybrid of Citrus triptera × C. sinensis) also increased regeneration by 4.7 times compared to explants transformed with a vector without the GRF–GIF insert. This shows that this approach can also be used to overcome recalcitrance in dicotyledonous species, in particular, in legumes.

In soybean, for example, 22 genes of the GmGRFs (Glycine max GRFs) family have been identified to date, localized on 14 chromosomes (Chen F. et al., 2019). Another family of transcription factors, WUSCHEL-related homeobox (WOX), is represented in soybean by 33 genes, and out of 19 soybean chromosomes, these genes are absent only on one chromosome, 16 (Hao et al., 2019). In this regard, experiments on QTL mapping of regeneration and transformation efficiency would help to find out which genes of these families of “global” transcription factors have the greatest effect on plant regeneration in culture. For example, the above-mentioned study on mapping a QTL in soybean that explains 26 % of the variability in somatic embryogenesis frequency (Song et al., 2010) indicates the presence of possible candidate genes on chromosome 6 in the region of the microsatellite marker Satt286 (physical position ~16,171,913 bp). One of the GRF family genes, the GmGRF5 gene (Glyma.06G134600), is located at a physical distance of ~5 Mb from the Satt286 marker, at a position of ~11,067,587 bp. Considering that the average genetic distance between markers on the used map was 28.4 cM, linkage between the Satt286 marker and the GmGRF5 gene can be assumed.

Genetic mapping is by no means the only way to identify morphogenetic regulators; today, multi-omics approaches are also used to search for them. For example, X. Liu et al. (2023) identified 446 key transcription factors regulating callus induction in wheat by combining three omics approaches at once: RNA-seq, ATAC-seq (Assay for Transposase-Accessible Chromatin using sequencing) and CUT&Tag (Cleavage Under Targets and Tagmentation). Based on the results of transcriptome profiling and analysis of the dynamics of epigenetic changes accompanying the process of regeneration from immature wheat embryo of Fielder variety, the authors identified two new genes, TaDOF5.6 and TaDOF3.4, the overexpression of which significantly increased the induction of callus and the efficiency of transformation in the wheat varieties Fielder, JM22 and Kenong 199.

Today, based on available resources, researchers have the opportunity to choose between a multi-omics approach to find factors influencing the efficiency of regeneration and transformation of the plants they work with, and the classical method of mapping these factors in segregating populations. The latter still seems less expensive, so studies on genetic mapping of regeneration and embryogenesis efficiency QTLs in recalcitrant species have obvious prospects.

## Conclusion

Annotated reference genomes available for many crop species, as well as modern genotyping and high-resolution genetic mapping capabilities, can significantly simplify the search for genes, the expression level or allelic polymorphism of which influences plant behavior in vitro.

## Conflict of interest

The authors declare no conflict of interest.
